# Supplementary material for: Evaluating the Growth of Ceria-Modified N-Doped Carbon-Based Materials and Their Performance in the Oxygen Reduction Reaction
Source: Nanomaterials (Basel). 2022 Sep 2;12(17):3057. doi: 10.3390/nano12173057 (PMC9457935; doi:10.3390/nano12173057)
Supplement: Supplementary file 1 [file nanomaterials-12-03057-s001.zip › nanomaterials-1837116-supplementary.pdf]

nanomaterials

Article

Evaluating the Growth of Ceria-Modified N-Doped Carbon-Based Materials and Their Performance in the Oxygen Reduction Reaction

Xin Wen, Ying Chang * and Jingchun Jia

Inner Mongolia Key Laboratory of Green Catalysis and Inner Mongolia Collaborative Innovation Center for Water Environment Safety, College of Chemistry and Environmental Science, Inner Mongolia Normal University, Hohhot 010022, China
* Correspondence: changying@imnu.edu.cn

Experimental Details

S1. Test Details

Slurry preparation: 5 mg catalyst is dissolved in 1mL nafion solution. The Nafion solution is prepared according to the ratio of nafion: ultrapure water: ethanol = 1:9:10. It is ultrasoniced for 30 minutes for standby. It is put into the refrigerator in time after use. It is ultrasoniced for at least 30 minutes before the next use.

Electrochemical test: Commercial Pt/C slurry drops 8 μL at one time on the surface of rotating disk electrode or rotating ring disk electrode with a pipette gun. The remaining catalyst slurry is evenly dropped on the electrode surface twice with a pipette gun, with a total of 16 μL. They were naturally evaporated at room temperature and used as working electrode, carbon rod as counter electrode and Hg/HgO as reference electrode to form a three-electrode system for ORR electrochemical test. The test was carried out using AutoLab electrochemical workstation (Metrohm, Switzerland) in constant temperature 25 °C, 0.1 M KOH solution and O_2 or Ar saturated atmosphere. In the test system, the glassy carbon electrode (diameter is 5 mm) coated with catalyst slurry is selected as the working electrode, the carbon rod (diameter is 5 mm) is used as the counter electrode, and the saturated calomel electrode is used as the reference electrode.

Methanol Tolerance Test: Methanol is added at a concentration of 1 M.

Zn-Air battery test: A total of 200 μL of catalyst slurry were added to the carbon paper-foam nickel composite matrix（1×1 cm^{-2}）four times, and then dried under the light as an air cathode; the polished Zn sheet is used as a metal anode; The electrolyte is a mixed solution of 6 M KOH and 0.2 M $Zn(Ac)_2$. CHI760E electrochemical workstation (Shanghai Chenhua Co., Ltd.) is used to test the charge discharge curve and EIS (Frequency range: 0.01-100000 Hz, Init E: 1.35V) of the battery and calculate the power density; CT2001A blue electric test system (Wuhan Blue Electric Electronics Co., Ltd.) was used to test the performance of self-made Zn-Air battery.

S2. Calculation of Transfer Electron Number and H_2O_2 Yield

K-L Curve FItting:

The K-L (Koutecky-Levich) equation is used to fit the LSV curve obtained by electrochemical test, so as to obtain the electron transfer number in the reaction process. The equation is as follows:

$$\frac{1}{\mathrm{j}}=\frac{1}{\mathrm{j_k}}+\frac{1}{\mathrm{j_d}}=\frac{1}{\mathrm{n}FkC_{O_2}}+\frac{1}{0.20\mathrm{n}FC_{O_2}D_{O_2}^{\frac{2}{3}}v^{-\frac{1}{6}}w^{\frac{1}{2}}} \quad \text{(S1)}$$

The total current density j includes the kinetic current density j_k and the limit diffusion current density j_d. n represents the total number of electrons transferred by each oxygen molecule, F is the Faraday constant, k is the reaction rate constant, C_{O_2} is the volume concentration of O_2, D_{O_2} is the diffusion coefficient of O_2, v represents the kinetic viscosity and w represents the angular velocity of the disk.

Calculation of RRDE Results:

H_2O_2 yield:

$$H_2O_2\%=200\times\frac{\frac{I_R}{N}}{\frac{I_R}{N}+I_D} \quad \text{(S2)}$$

Electron transfer number：

$$n=4\times\frac{I_D}{I_D+\frac{I_R}{N}} \quad \text{(S3)}$$

Where I_R represents the ring current, I_D represents the disk current, and N is the current collection efficiency of Pt ring electrode.

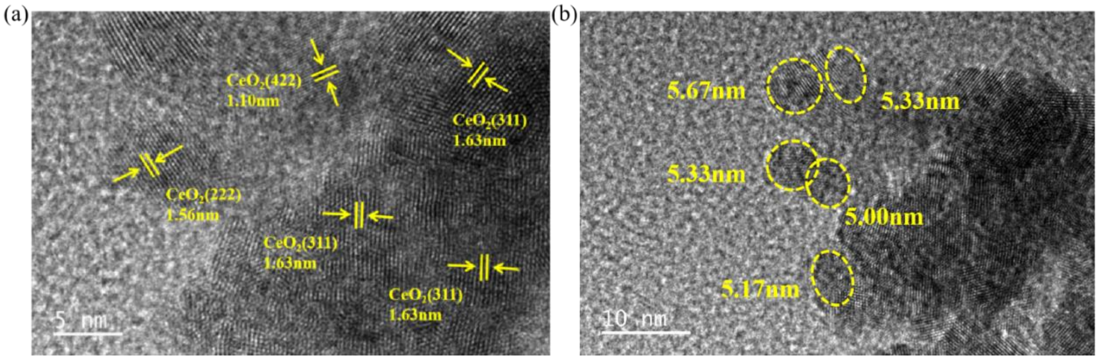

Figure S1. (a), (b) TEM diagrams of CeO_2–CN–800.

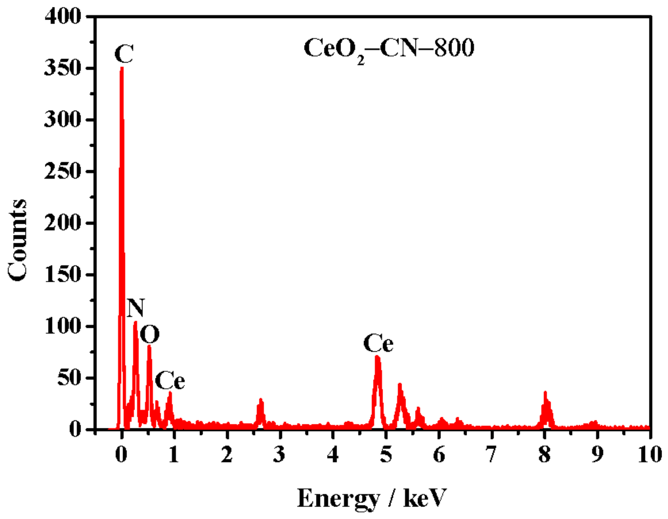

Figure S2. EDS diagram of CeO2–CN–800.

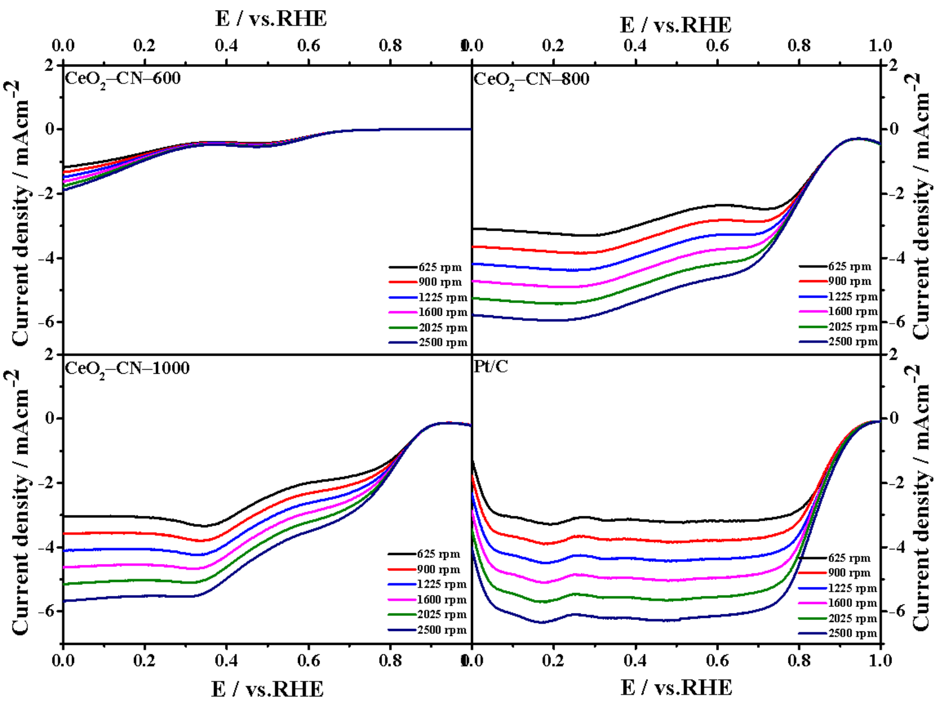

Figure S3. LSV curves of CeO2–CN–600, CeO2–CN–800, CeO2–CN–1000 and Pt/C.

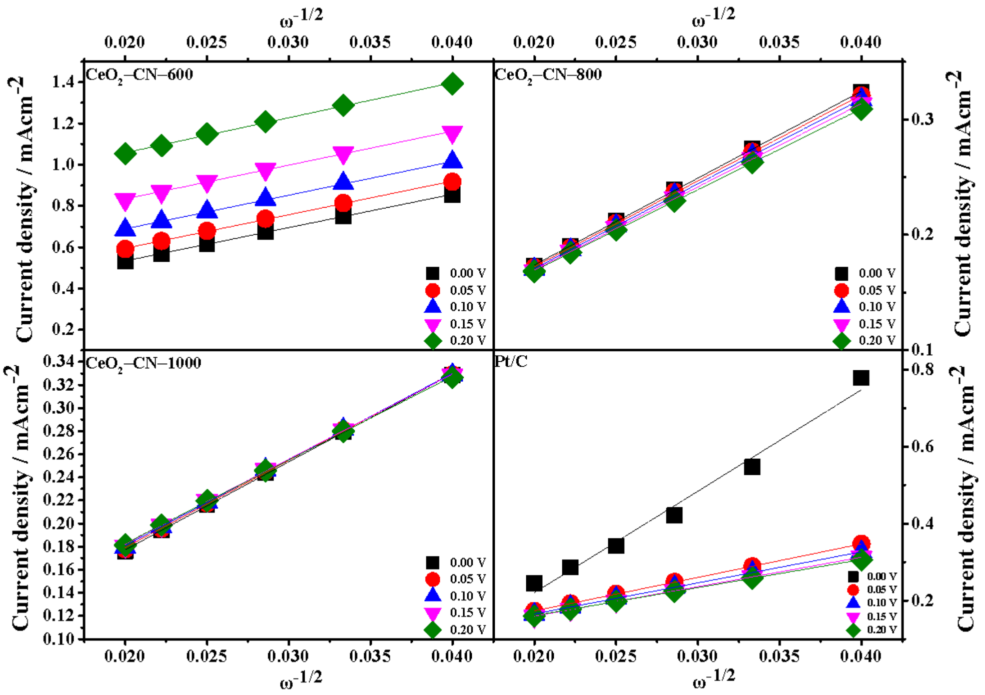

Figure S4. K-L curves of CeO2–CN–600, CeO2–CN–800, CeO2–CN–1000 and Pt/C.

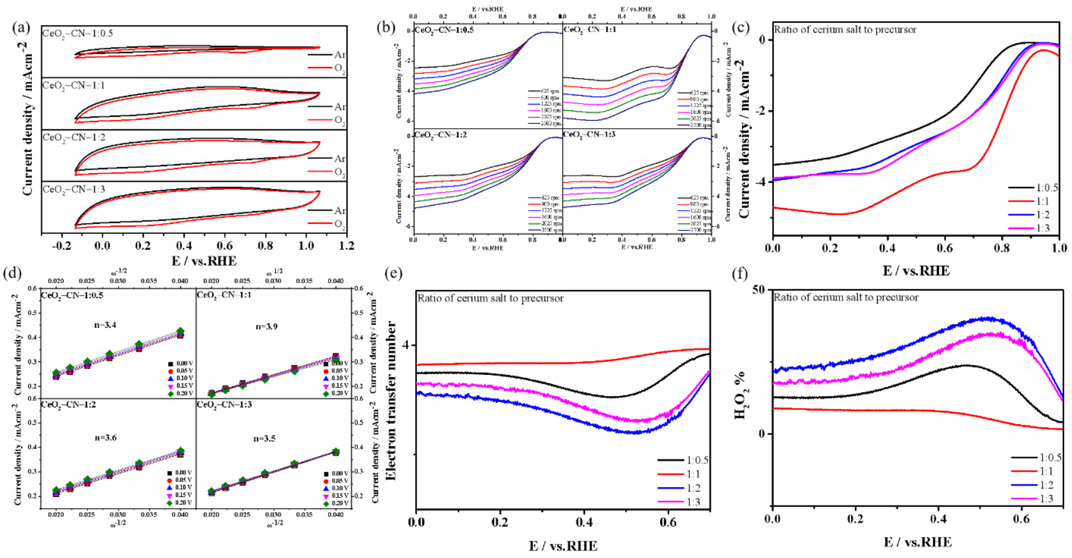

Figure S5. (a) CV diagrams, (b) LSV diagrams, (c) LSV curves comparison diagram, (d) K-L curves, (e) Electron transfer number, and (f) Hydrogen peroxide yield of different ratios of cerium salt to precursor.

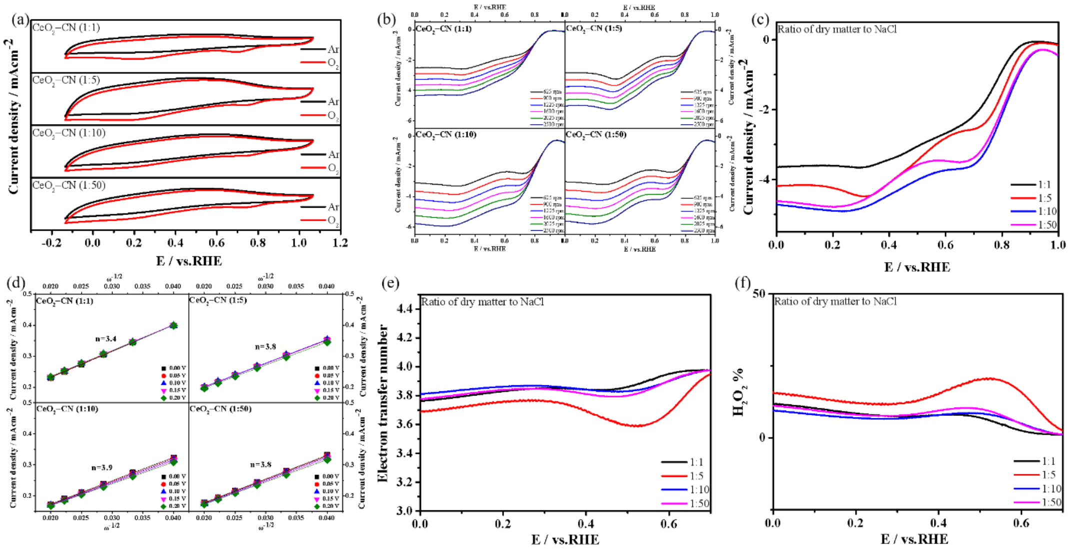

Figure S6. (a) CV diagrams, (b) LSV diagrams, (c) LSV curves comparison diagram, (d) K-L curves, (e) Electron transfer number, and (f) Hydrogen peroxide yield of different ratios of dry matter to NaCl.

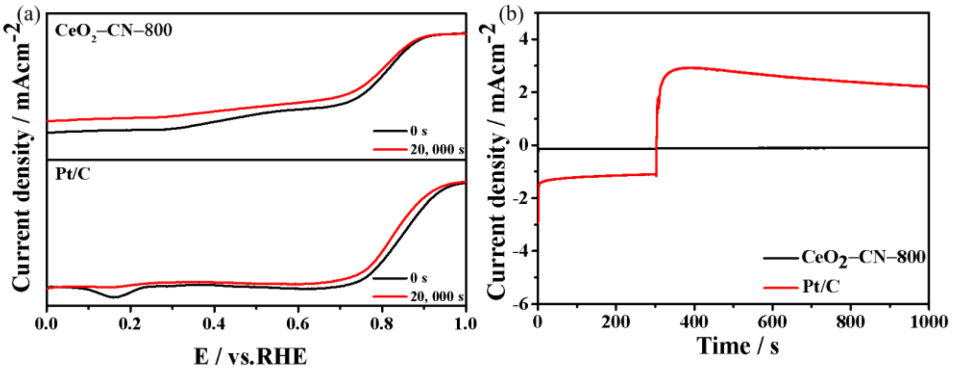

Figure S7. (a) LSV diagrams before and after i-t stability test, (b) i-t curves before and after adding methanol of CeO_2–CN–800 and Pt/C.

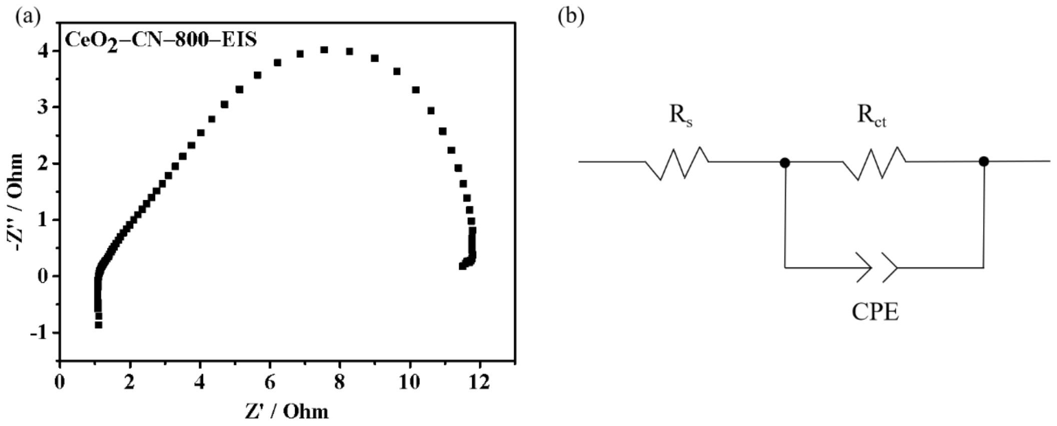

Figure S8. (a) Electrochemical impedance spectrogram and (b) its equivalent circuit diagram of CeO_2–CN–800 in zinc-air battery.

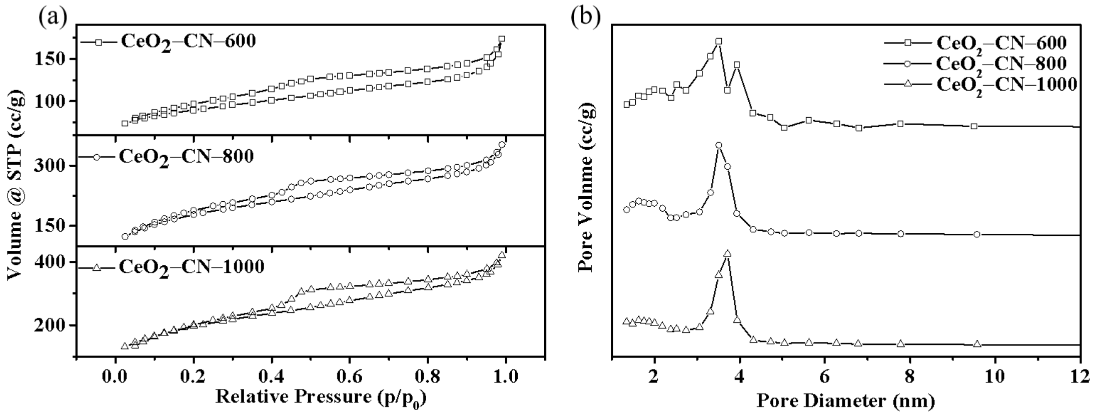

Figure S9. (a) Nitrogen adsorption-desorption isotherm, (b) pore size distribution curve of CeO_2–CN–600, CeO_2–CN–800, CeO_2–CN–1000.

Table S1. Summary of some recently reported CeO_2 based electrocatalysts in alkaline electrolyte.

Catalyst.	E_o (V vs.RHE)	$E_{1/2}$ (V vs.RHE)	Reference
Co_3O_4–CeO_2/KB	-	0.83	[1]
CeO_2@N–C–900	1.00	0.90	[2]
CeO_2 hollow spheres	0.84	0.76	[3]
$NiCo_2O_4$/CeO_2	0.84	0.69	[4]
Pd–$CeO_{2\text{-}NR}$/C	0.97	0.87	[5]
4.8% Ce–MnO_2/C	0.87	0.78	[6]
CeO_2@NC–900	0.90	0.85	[7]
0.5 Co–NC–CeO_2	0.87	0.81	[8]
Pd_3Ag_1/meso–CeO_2	0.97	0.86	[9]
CeO_2/VC (80 wt%)	0.76	0.63	[10]
CeO_2/$MnWO_4$–2	-	0.60	[11]
CeO_2–CN–800	0.90	0.84	This work

Table S2. Calculation of average crystallite size from XRD results using Scherrer formula.

2 Theta	FWHM	Crystallite Size D (nm)	Average D (nm)
28.37892	1.45384	5.64	
32.86566	1.48689	5.57	
47.19659	1.42589	6.08	
56.07876	1.58726	5.67	5.21
76.45285	71.70431	0.14	
76.45285	1.64782	6.14	
78.67725	1.42167	7.23	

Table S3. BET surface area, pore volume and pore size of CeO_2–CN–600, CeO_2–CN–800, CeO_2–CN–1000.

Sample	BET surface area (m^2/g)	Pore volume (cc/g)	Pore size (nm)
Ce_2O–CN-600	291.1	0.27	3.69
Ce_2O–CN-800	607.6	0.54	3.58
Ce_2O–CN-1000	683.9	0.65	3.81

Table S4. Elemental analysis results of Ce_2O-CN-800.

	C (wt %)	N (wt %)	O (wt %)	Ce (wt %)
Ce_2O–CN-800	63.15	3.96	23.45	9.43

Table S5. Deconvolution results of N 1s XPS spectra of CeO_2–CN–600, CeO_2–CN–800, CeO_2–CN–1000.

Sample	pyridinic-N (rel. %)	pyrrolic-N (rel. %)	graphitic-N (rel. %)
Ce_2O-CN-600	76.59	23.41	0
Ce_2O-CN-800	86.20	11.17	2.63
Ce_2O-CN-1000	28.67	71.33	0

References

1. Liu, K.; Huang, X.; Wang, H.; Li, F.; Tang, Y.; Li, J.; Shao, M., Co_3O_4-CeO_2/C as a highly active electrocatalyst for oxygen reduction reaction in Al-air batteries. *ACS Applied Materials* & *Interfaces* **2016,** *8* (50), 34422–34430.
2. Yu, Y.; Gao, L.; Liu, X.; Wang, Y.; Xing, S., Enhancing the catalytic activity of zeolitic imidazolate framework-8-derived N-doped carbon with incorporated CeO_2 nanoparticles in the oxygen reduction reaction. *Chemistry* **2017,** *23* (44), 10690–10697.
3. Ghosh, D.; Parwaiz, S.; Mohanty, P.; Pradhan, D., Tuning the morphology of CeO_2 nanostructures using a template-free solvothermal process and their oxygen reduction reaction activity. *Dalton Trans.* **2020,** *49* (48), 17594–17604.
4. Wang, J.; Dong, X.; Yang, J.; Zhu, L.; Zeng, W.; Wang, J., Facile hydrothermal synthesis of 3D flower-like $NiCo_2O_4$/CeO_2 composite as effective oxygen reduction reaction catalyst. *J. Mater. Sci. : Mater. Electron.* **2020,** *31* (19), 16600–16608.
5. Meléndez‐González, P. C.; Sánchez‐Castro, E.; Alonso‐Lemus, I. L.; Pérez‐Hernández, R.; Escobar‐Morales, B.; Garay‐Tapia, A. M.; Pech‐Rodríguez, W. J.; Rodríguez‐Varela, J., Bifunctional Pd‐CeO_2 nanorods/C nanocatalyst with high electrochemical stability and catalytic activity for the ORR and EOR in alkaline media. *ChemistrySelect* **2020,** 5 (44), 14032–14040.
6. Sun, S.; Xue, Y.; Wang, Q.; Huang, H.; Miao, H.; Liu, Z., Cerium ion intercalated MnO_2 nanospheres with high catalytic activity toward oxygen reduction reaction for aluminum-air batteries. *Electrochim. Acta* **2018,** *263*, 544–554.
7. Kang, Y.; Wang, W.; Li, J.; Mi, Y.; Gong, H.; Lei, Z., 3D rosa centifolia-like CeO_2 encapsulated with N-doped carbon as an enhanced electrocatalyst for Zn-air batteries. *J. Colloid Interface Sci.* **2020,** *578*, 796–804.
8. Wang, X.; Xu, J.; Wu, Z.; Zhi, M.; Hong, Z.; Huang, F., Complexing‐coprecipitation method to synthesize catalysts of cobalt, nitrogen‐doped carbon, and CeO_2 nanosheets for highly efficient oxygen reduction. *ChemNanoMat* **2019,** *5* (6), 831–837.
9. Sridharan, M.; Maiyalagan, T., Enhanced oxygen reduction activity of bimetallic Pd–Ag alloy-supported on mesoporous cerium oxide electrocatalysts in alkaline media. *New J. Chem.* **2021,** *45* (47), 22181–22192.
10. Hota, I.; Debnath, A. K.; Muthe, K. P.; Varadwaj, K. S. K.; Parhi, P., A synergistic approach of Vulcan carbon and CeO_2 in their composite as an efficient oxygen reduction reaction catalyst. *J. Appl. Electrochem.* **2020,** *50* (10), 1069–1077.
11. Sridharan, M.; Maiyalagan, T., Synergistically enhanced electrocatalytic activity of cerium oxide/manganese tungstate composite for oxygen reduction reaction. *J. Mater. Sci. : Mater. Electron.* **2022,** *33* (12), 9538–9548.
